# Medication risk management and health equity in New Zealand general practice: a retrospective cross-sectional study

**DOI:** 10.1186/s12939-021-01461-y

**Published:** 2021-05-11

**Authors:** Sharon Leitch, Jiaxu Zeng, Alesha Smith, Tim Stokes

**Affiliations:** 1 Department of General Practice and Rural Health, Otago Medical School – Dunedin Campus, University of Otago, Dunedin, New Zealand; 2 Department of Preventive and Social Medicine, Otago Medical School – Dunedin Campus, University of Otago, Dunedin, New Zealand; 3 School of Pharmacy, University of Otago, Dunedin, New Zealand

**Keywords:** Ethnicity, Equity, Decision support, General practice, Harm

## Abstract

**Background:**

Despite an overt commitment to equity, health inequities are evident throughout Aotearoa New Zealand. A general practice electronic alert system was developed to notify clinicians about their patient’s risk of harm due to their pre-existing medical conditions or current medication. We aimed to determine whether there were any disparities in clinician action taken on the alert based on patient ethnicity or other demographic factors.

**Methods:**

Sixty-six New Zealand general practices from throughout New Zealand participated. Data were available for 1611 alerts detected for 1582 patients between 1 and 2018 and 1 July 2019. The primary outcome was whether action was taken following an alert or not. Logistic regression was used to assess if patients of one ethnicity group were more or less likely to have action taken. Potential confounders considered in the analyses include patient age, gender, ethnicity, socio-economic deprivation, number of long term diagnoses and number of long term medications.

**Results:**

No evidence of a difference was found in the odds of having action taken amongst ethnicity groups, however the estimated odds for Māori and Pasifika patients were lower compared to the European group (Māori OR 0.88, 95 %CI 0.63–1.22; Pasifika OR 0.88, 95 %CI 0.52–1.49). Females had significantly lower odds of having action taken compared to males (OR 0.76, 95 %CI 0.59–0.96).

**Conclusions:**

This analysis of data arising from a general practice electronic alert system in New Zealand found clinicians typically took action on those alerts. However, clinicians appear to take less action for women and Māori and Pasifika patients. Use of a targeted alert system has the potential to mitigate risk from medication-related harm. Recognising clinician biases may improve the equitability of health care provision.

**Supplementary Information:**

The online version contains supplementary material available at 10.1186/s12939-021-01461-y.

## Background

Health inequities are defined as differences in health outcomes or risks to health between peoples of different social advantage [1]. Te Tiriti O Waitangi (The Treaty of Waitangi), the founding constitutional document of Aotearoa New Zealand (NZ), upholds the ideals of equity and protection of Māori (the Indigenous people) [2–4].

New Zealand provides universal cover for most health services, including hospital-based inpatient and outpatient care [5]. Primary healthcare is delivered in community-based general practices. General practice services and medication for children under the age of 14 years are fully subsidised. However, most other patients co-pay for primary healthcare (typically $13 -$35 USD) and pay a small prescription charge per medication ($3.50 USD). Annual out-of-pocket health spending per capita is $520 USD, accounting for around 12 % of New Zealand total health spending [6]. In comparison, the median weekly income is $455 USD; Māori and Pasifika (people living in NZ who identify as Pacific peoples) have lower median weekly incomes than people of other ethnicities [7].

Systemic racism is widespread in the New Zealand health system [8]. Despite ambitious national goals to “improve, promote and protect the health and wellbeing of New Zealanders” [9], healthcare inequity persists for Māori and Pasifika [10]. People of Māori and Pasifika ethnicity and people who experience socioeconomic deprivation, have excessively high adverse event rates, including premature mortality, injury, disability, and healthcare-related harms [10–15]. These groups experience under-prescribing of appropriate medications, higher prescribing of inappropriate medications [16], and higher rates of polypharmacy [17, 18].

Computerised decision support tools can help improve the quality and safety of prescribing by identifying and alerting clinicians to potentially dangerous prescribing actions [19–22]. Conporto Health Event Detection & Mitigation (Conporto EDM) is an automated alert system that detects whether general practice patients are at high risk of medical harm due to their medical conditions, medications, or for want of mitigating preventative action [23]. Events in this system consist of 10 pre-specified conditions (Table 1). The system is triggered by activities such as making an appointment, or a prescription request. Clinicians are informed at the start of each session which of their patients to be seen are at increased risk of harm via secure email, with detailed information sent to the electronic health record “Inbox”. The “Inbox” is a portal containing all messages relevant to that patient, including laboratory results, radiology results, correspondence from secondary care, and Conporto EDM alerts. Clinicians have full discretion as to whether they act on the Conporto EDM alerts. Patients are not advised of the alerts unless informed by their clinician. In an attempt to avoid alert fatigue, clinicians are only notified of each triggered Conporto EDM alert once every three months. Therefore, if a Conporto EDM alert is triggered by the patient making an appointment or requesting more medication within three months since the last alert, the clinician will not be notified of those alerts.

**Table 1:** Conporto EDM alerts

	Alert	Description
1	Allopurinol > 200 mg, eGFR < 30	Allopurinol prescribed at a dose of > 200 mg/day to a patient with chronic renal insufficiency (eGFR < 30 mL/min/1.73 m^2^)^a^
2	Macrolide & simvastatin	Prescription for an macrolide antibiotic, with a co-prescription for simvastatin
3	Bupropion epilepsy	Bupropion (Zyban) prescribed to a patient with epilepsy
4	Metformin eGFR < 30	Metformin prescribed to a patient with renal insufficiency where the eGFR is < 30 mL/min/1.73 m^2^
5	MTX no Folic acid	Prescription of methotrexate, without a co-prescription for folic acid
6	NSAID eGFR < 45	Prescription of a NSAID^b^, in a patient with chronic renal insufficiency (eGFR < 45 mL/min/1.73 m^2^)
7	NSAID, Ulcer, no PPI	Prescription of a NSAID, without co-prescription for an proton-pump inhibitor to a patient with a history of peptic ulceration
8	PDEi & Nitrite	Prescription of a phosphodiesterase type-5 inhibitor, with a co-prescription for a nitrate
9	Valproate F epilepsy	Prescription of sodium valproate to a female aged 10–59 years with a diagnosis of epilepsy, without history of hysterectomy
10	Valproate F	Prescription of sodium valproate to a female aged 10–59 years, without history of hysterectomy OR epilepsy

^a^*eGFR *estimated glomerular filtration rate, specified here as having been calculated with the Cockcroft-Gault equation

^b^*NSAID *Non-steroidal anti-inflammatory drug, e.g. aspirin, ibuprofen, diclofenac, etc.

Preliminary analysis of Conporto EDM data from 1 March – 31 October 2018 was undertaken by Conporto Health [23]. This suggested that although general practitioners did generally take action following an event alert notification, when analysed by individual harm event they appeared less likely to take action for Māori and Pasifika patients [23]. However, important confounders were not adjusted for in those analyses. Also, action rates were evaluated by individual event, even though four of the event groups were too small to make statistical inferences when broken down by ethnicity. We therefore re-examined the association between the actions clinicians took after receiving an event alert and patient ethnicity to determinate robustness of the earlier findings. We did this by grouping all alerts to look at action taken as a whole rather than by individual event, and adjusting for a set of important confounders.

The aim of this study is to determine whether there were any disparities in clinician action taken (versus no action) following an alert based on patient ethnicity or other demographic factors.

## Methods

General practices were recruited from all regions of New Zealand, from those participating in the Conporto Health Look-Up programme (an online platform presenting an integrated summary patient record between healthcare providers). Sixty-six practices signed a consent form to participate in the Conporto EDM proof-of-concept trial. Study participants were patients attending those clinics; individual patient consent was not obtained. Ethical approval for this secondary data review was obtained from the University of Otago Human Ethics Committee (HD19/061). The project was also reviewed by the Ngāi Tahu research consultation committee.

### Derivation of study alerts

During the proof-of-concept trial, patient clinical notes were retrospectively reviewed to see whether clinicians took action or not after receiving an alert. The review was undertaken initially by a computer programme which scanned the notes and could determine if action was taken depending on the text, e.g. “stop metformin”; further review was undertaken by a GP and a pharmacist if the results from the computer review were ambiguous.

**Fig. 1:**
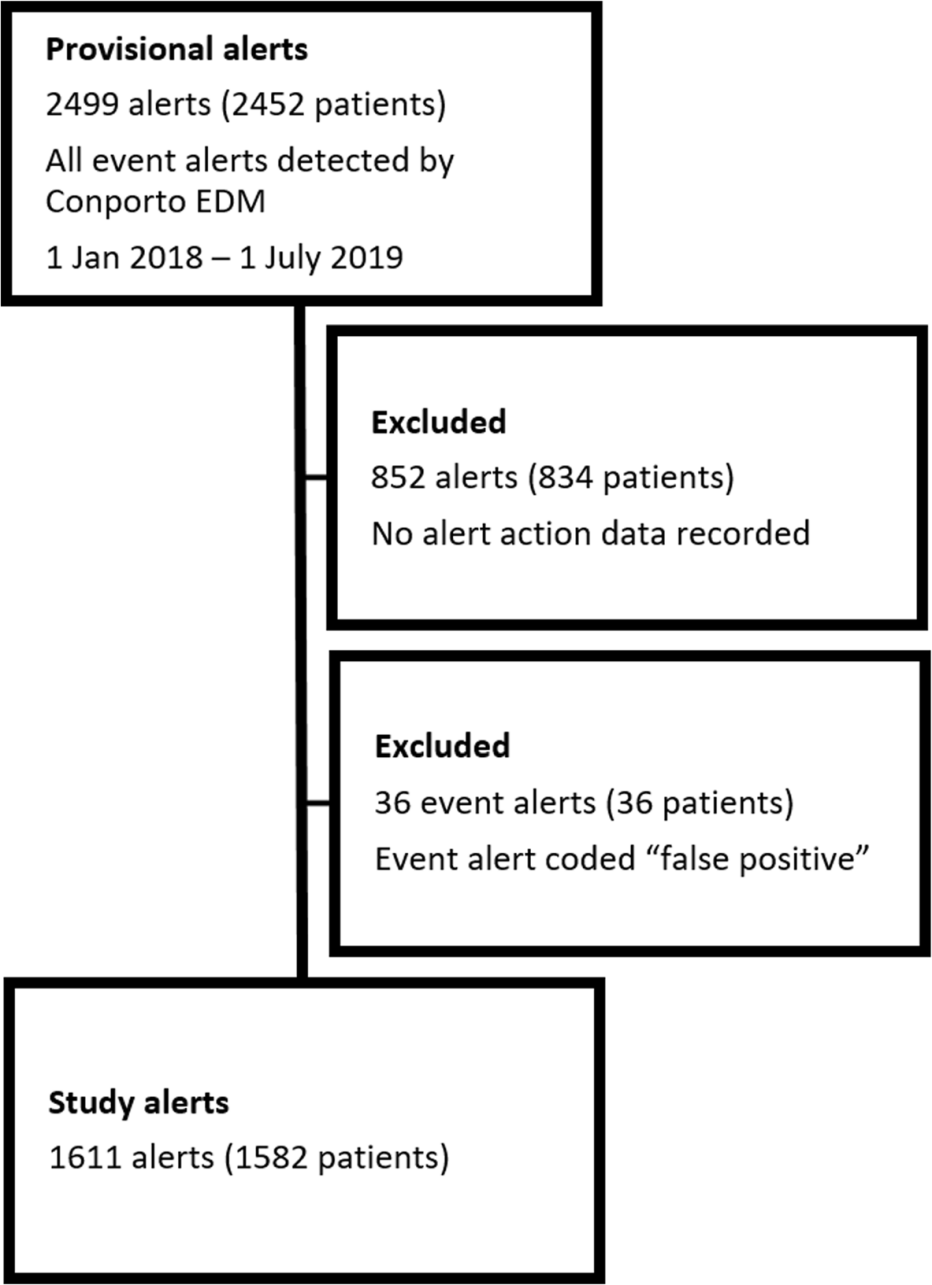
Derivation of study data

Conporto Health provided information for all alerts recorded in Conporto EDM between 1 January 2018 and 1 July 2019, with patient information retrieved from the general practice records secondary to event data. Figure 1 illustrates the steps for the identification of the study alerts. 2499 event alerts were detected within the study period. We excluded 852 alerts where there was no action data recorded; i.e. the medical records had not been reviewed to determine whether action was taken or not. A further 36 events were excluded which had been coded as “false positive” during the proof-of-concept trial, i.e. after checking the medical records, the initial alert was found to be incorrect. Alert data was linked with patient general practice information held by Conporto, extracted from study general practices using the electronic health record.

### Primary outcome

The primary outcome measure was whether clinicians took action or not after receiving a Conporto EDM alert. All alert types were analysed as one group. Alert consequences were categorised into “action” or “no action taken,” as determined by Conporto reviewers during the preliminary analysis period.

### Covariates

Ethnicity is self-identified in New Zealand, and people can identify with more than one ethnic group [24]. Prioritised ethnicity was used in concordance with standard New Zealand health and disability sector use [24]. Patients were categorised to one of the following five categories European, Māori, Pasifika, Asian and Other. “Other” ethnicity in this dataset includes people of Middle Eastern, Latin American and African ethnicities. Deprivation in New Zealand is assessed by geographical meshblock, by combining census data parameters including income, home ownership, and employment (NZDep13) [25]. Patients were assigned to the deprivation groups according to their NZDep13 score, in which deprivation increases by group number. Group 1 (scores 1 and 2) represents the least deprived area; groups 2 (score 3 and 4), 3 (5 and 6), and 4 (7 and 8) are increasingly deprived, while group 5 (9 and 10) represents the most deprived.

In New Zealand, clinicians highlight long-term diagnoses and long-term medications in their patient’s electronic health record in order to provide best practice care and facilitate communication between healthcare providers. Typically, long-term diagnoses refer to conditions that the patient receives active treatment for, and serious historic diagnoses. Long-term medications are those which the patient is prescribed regularly (e.g. every three months).

Demographic data (age at the time of the GP appointment, gender, ethnicity and socioeconomic deprivation) and clinical data (number of long-term diagnoses and number of long-term medications) were extracted from the electronic health records. Age, number of long-term diagnoses and long-term medications were treated as categorical variables. Age was divided into three clinically meaningful groups – those aged 1–49 years, 50–74 years and 75 years and older. Numbers of diagnoses and medications are grouped into three clinically meaningful groups; 1–5 long-term diagnoses or long-term medications, 6–10, and 11 or more.

### Missing data

Complete data was available for 1235/1611 (76.7 %) events. Information on covariates was missing if it was absent in the general practice records. The covariate with the most missing data was long-term medications (301/1611 events, 18.7 %), followed by socio-economic depreivation (181/1611, 11.3 %) and long-term diagnoses (144/1611, 8.9 %).

### Statistical analyses

Logistic regression with robust standard error was used to investigate if the actions taken differed across ethnicity groups. Robust standard error allows correlations between the events reported from the same patient. We considered potential confounding by age, gender, socio-economic deprivation, number of long-term diagnoses, and number of long-term prescriptions. Each covariate was initially fitted separately, then with ethnicity. The final model included all covariates.

Unadjusted and adjusted odd ratios along with 95 % CI were reported for each covariate. Finally, the EDM alerts were analysed by event type. The number and proportion of notified alerts were reported and of those alerted events, those of actioned events were also reported. Complete-case analysis was used for handling missing data and was performed based on 1235 events. All statistical analyses were performed using Stata software version 15.1 [26].

## Results

Table 2 shows events by patient demographic and clinical covariates, by action (whether action was taken or not).

**Table 2:** Table of events by patient demographic and clinical covariates

Variable	No Action *n* = 820 (50.9 %)^a^	Action *n* = 791 (49.1 %)^a^	Total *n* = 1611 (100 %)^b^
**Age** in years			
1–49	166 (55.7)	132 (44.3)	298 (18.5)
50–74	351 (47.4)	389 (52.6)	740 (45.9)
75 or more	303 (52.9)	270 (47.1)	573 (35.6)
Missing	0	0	0
**Gender**			
Male	274 (45.1)	333 (54.9)	607 (37.7)
Female	546 (54.4)	458 (45.6)	1004 (62.3)
Missing	0	0	0
**Prioritised Ethnicity**			
European	577 (50.8)	560 (49.3)	1137 (70.6)
Māori	128 (53.1)	113 (46.9)	241 (15.0)
Pasifika	40 (48.8)	42 (51.2)	82 (5.1)
Asian	42 (41.6)	59 (58.4)	101 (6.3)
Other	33 (66.0)	17 (34.0)	50 (3.1)
Missing	0	0	0
**Deprivation**^c^			
1	103 (48.6)	109 (51.4)	212 (13.2)
2	119 (52.2)	109 (47.8)	228 (14.2)
3	159 (53.4)	139 (46.6)	298 (18.5)
4	172 (49.7)	174 (50.3)	346 (21.5)
5	167 (48.3)	179 (51.7)	346 (21.5)
Missing	100 (55.3)	81 (44.8)	181 (11.3)
**Long-term diagnoses**			
1–5	376 (52.5)	340 (47.5)	716 (44.5)
6–10	287 (49.9)	288 (50.1)	575 (35.7)
11 or more	75 (42.6)	101 (57.4)	176 (10.9)
Missing	82 (56.9)	62 (43.1)	144 (8.9)
**Long-term medications**			
1–5	281 (53.3)	246 (46.7)	527 (32.7)
6–10	244 (47.0)	275 (53.0)	519 (32.2)
11 or more	112 (42.4)	152 (57.6)	264 (16.4)
Missing	183 (60.8)	118 (39.2)	301 (18.7)

^a^Action Columns show number, row percentage

^b^Total Column shows number, column percentage for each section

^c^Deprivation: 1 represents the least socioeconomically deprived, 5 the most deprived

Around half of alerts results in an action (791/1611, 49.1 %). Most alerts occurred in patients aged at least 50 years old (1313/1611, 81.5 %) and female (1004/1611, 62.3 %). There was no clear pattern of action taken by age group. Females had proportionally less action taken than males (female action taken 458/1004, 45.6 %; male 333/607, 54.9 %).

NZ European ethnicity constituted 70.6 % of the sample (1137/1611), Māori 241 (15.0 %), Pasifika 82 (5.1 %), Asian 101 (6.3 %) and Other 50 (3.1 %). Patients of Asian ethnicity were proprtionally most likely to have action taken (59/101, 58.4 %), and patients of Other ethnicity were least likely to have action taken (17/50, 34.0 %). More than 40 % of the sample lived in areas of high deprivation (NZDep13 quintile 4 or 5 = 692/1611, 43.0 %). There was no clear pattern in the proportion of action taken by deprivation.

The median number of long term diagnoses was 6 (IQR 3–8), and the median number of long-term medication 7 (IQR 4–10). There appeared to be a positive trend towards more action taken with increasing number of both long-term diagnoses and long-term medications.

Table 3 shows that the adjusted odds of having action taken for Māori patients was 0.88 (95 %CI 0.63–1.22) times that of European patients. Similarly, Pasifika ethnicity was associated with a reduced adjusted odds of receiving actions (OR = 0.88, 95 %CI 0.52–1.49) compared to Europeans. Although the estimated odds suggest that Māori and Pasifika patients were less likely to be treated, the results are not statistically significant. In addition, patients of Asian ethnicity had increased odds of having action taken (OR 1.39, 95 % CI 0.86–2.23), however the association was not statistically significant.

**Table 3:** The unadjusted and adjusted odds ratios of action for all events taken, by patient characteristics and clinical covariates

	Unadjusted		Adjusted	
**Variable**	**OR (95 % CI)**	***p*** value	**OR (95 % CI)**	***p*** value
**Age in years**				
1–49	1 [Reference]	-	1 [Reference]	-
50–74	1.39 (1.06–1.83)	0.016	1.28 (0.92–1.79)	0.144
75 or more	1.12 (0.85–1.48)	0.428	1.15 (0.80–1.67)	0.446
**Gender**				
Male	1 [Reference]	-	1 [Reference]	-
Female	0.69 (0.56–0.85)	< 0.001	0.76 (0.59–0.96)	0.023
**Ethnicity**				
European	1 [Reference]	-	1 [Reference]	-
Māori	0.91 (0.69–1.20)	0.505	0.88 (0.63–1.22)	0.446
Pasifika	1.08 (0.69–1.69)	0.731	0.88 (0.52–1.49)	0.646
Asian	1.45 (0.96–2.19)	0.079	1.39 (0.86–2.23)	0.178
Other	0.53 (0.29–0.96)	0.037	1.09 (0.43–2.79)	0.851
**Deprivation**^a^				
1	1 [Reference]	-	1 [Reference]	-
2	0.87 (0.60–1.26)	0.450	0.82 (0.54–1.23)	0.332
3	0.83 (0.58–1.18)	0.288	0.79 (0.54–1.16)	0.231
4	0.96 (0.68–1.35)	0.796	0.99 (0.68–1.45)	0.959
5	1.01 (0.72–1.43)	0.942	0.98 (0.66–1.45)	0.918
**Long-term diagnoses**				
1–5	1 [Reference]	-	1 [Reference]	-
6–10	1.11 (0.89–1.38)	0.353	1.04 (0.80–1.35)	0.792
11 or more	1.49 (1.07–2.08)	0.019	1.31 (0.87–1.96)	0.193
**Long-term medications**				
1–5	1 [Reference]	-	1 [Reference]	-
6–10	1.29 (1.01–1.64)	0.042	1.16 (0.88–1.52)	0.283
11 or more	1.55 (1.15–2.09)	0.004	1.25 (0.89–1.77)	0.201

^a^Deprivation: 1 represents the least socioeconomically deprived, 5 the most deprived

Women had reduced odds of having action taken compared to men in both the unadjusted and adjusted models. After adjusting for confounding, the odds ratio for women having action taken for an alert was 0.76 (95 %CI 0.59–0.96). There was no association found between action taken and age, social deprivation, number of long-term diagnoses, or number of long-term medications.

Table 4 shows that just under half of the events were actioned overall (791/1611, 49.1 %). The majority of events were notified (1358/1611, 84.3 %). Of those notified, 58.2 % (791/1358) were actioned. The most common event detected was co-prescription of a macrolide antibiotic and simvastatin. This accounted for more than one quarter of events (425/1611, 26.4 %). The least common event was a prescription for buproprion in a patient diagnosed with epilepsy (4/1611, 0.3 %). Excluding buproprion, notification rates ranged from 98.2 % (160/163 females of childbearing age prescribed sodium valporate for epilepsy) to 55.8 % (24/43 patients with low renal function who were prescribed a high dose of allopurinol). Clinicians proportionally took the most action for patients who were taking methotrexate but not folic acid (98/155, 63.2 %), and (excluding buproprion) the least action for females of childbearing age taking sodium valproate for epilepsy (48/163, 29.5 %). Individual event action rates (excluding buproprion) following notification ranged from 30.0 − 87.5 %.

**Table 4:** All Conporto EDM Alerts

Alert	N (% of all events)	Notified (% of event)	Actioned (% of notified)
Macrolide & Simvastatin	425 (26.4)	363 (85.4)	239 (65.8)
NSAID eGFR < 45	372 (23.1)	302 (81.2)	165 (54.6)
Meformin eGFR < 30	187 (11.6)	161 (86.1)	113 (70.2)
Valproate F	178 (11.1)	157 (88.2)	63 (40.1)
Valproate F epilepsy	163 (10.1)	160 (98.2)	48 (30.0)
MTX no folic acid	155 (9.6)	118 (76.1)	98 (83.1)
PDEi & Nitrite	51 (3.2)	44 (86.3)	29 (65.9)
Allopurinol > 200 mg eGFR < 30	43 (2.7)	24 (55.8)	21 (87.5)
NSAID, ulcer, no PPI	33 (2.1)	25 (75.8)	15 (60.0)
Buproprion epilepsy	4 (0.3)	4 (100)	0 (0)
**Total**	**1611 (100)**	**1358 (84.3)**	**791 (58.2)**

## Discussion

### Summary of findings

We found no evidence supporting the assertion that Māori or Pasifika ethnicity groups are associated with lower odds of clinicians taking action after an alert based on the reported confidence intervals. However, the estimated odds ratios do suggest that Māori or Pasifika ethnicity is associated with lower odds of clinicians taking action after an alert. Women had nearly double the number of alerts compared to men, which is consistent with the fact women see their GPs more frequently than men, even after excluding consultations relating to gynaecological and obstetric conditions [27]. Our study suggests that females were less likely to have action taken compared to males following an alert. Women have a long history of experiencing inequitable health care compared to men, such as receiving less pain relief for similar levels of acute and chronic pain [28, 29]. This may be attributable to the status of women in society; addressing gender equality is considered an important factor in improving women’s health [30].

### Strengths and limitations

This paper provides a snapshot of high-needs general practice patients in New Zealand, as well as some of the risks they are exposed to while receiving routine healthcare. This study had a wide geographical spread of patients, and an ethnic distribution profile similar to the New Zealand population, although the study had a lower proportion of Asian patients and a higher proportion of Other ethnicities [31]. A weakness of this study is that it could be underpowered to detect differences by ethnicity. Also, one quarter of participants had at least one missing covariate, and thus were not included in the analysis.

### Comparison with existing literature

The underlying premise of this work was a rich literature demonstrating increased risk of harm and unfair treatment of people based on ethnicity. This is well documented for Māori and Pasifika patients [8, 10]. Migrants and people who don’t speak English face additional challenges in a healthcare setting due to cultural and language barriers [32, 33]. In addition, preliminary review of these data led us to anticipate differences in clinician action based on ethnicity.

Our findings suggest patient gender is associated with whether general practice clinicians take action after receiving an alert. It is possible that patient ethnicity also has some effect, although our results are not statistically significant. While other factors may be at play, implicit associations of gender and ethnicity can play a role in medical judgement and result in biased provision of care [34–37].

### Implications for health policy

As the proportion of older patients increases in New Zealand general practice, so too do their numbers of long-term conditions and long-term medications [18, 38]. The burden of multimorbidity is known to be particularly high for Māori and Pasifika patients [39]. These factors add to the complexity of general practice consultations [40]. Targeted alert systems can help busy general practitioners identify patients at greatest risk of experiencing medication-related harm, and take actions to mitigate those risks [19, 41]. Clinicians in this study took action following receipt of targeted event alerts more often than not. Promoting use of such a system has the potential to reduce medication-related harm in general practice.

Inequitable care is evident throughout the New Zealand health system [4, 10]. The causes for this are multifactorial; no doubt racism and sexism contribute to health inequities, adverse patient experiences and negative health outcomes [42, 43]. While addressing these issues at a system level is important [10], this paper focussed on the action of individual clinicians. Training clinicians to speak up against racism and sexism, as well as recognise their own implicit biases, may help reduce inequities based on those characteristics [34, 44–46].

## Conclusions

This analysis of data arising from a general practice electronic alert system in New Zealand assessed whether clinicians took action on those alerts. Clinicians typically did take action. Our study has found no evidence to support the assertion that Māori and Pasifika ethnicity are associated with lower odds of having action taken on an alert, although the adjusted odds ratios suggest these ethnicity groups are associated with a lower odds, and therefore future studies would benefit from larger samples to investigate this research question further. Female sex is also associated with lower odds of having action taken. Recognising clinician biases may improve the equitability of health care provision.

## Supplementary Information

**Additional file 1.**

## Data Availability

The anonymised datasets analysed during the current study are attached as a supplementary file.

## Abbreviations

Conporto EDM: Conporto Health Event Detection & Mitigation system
GP: General practitioner
NZ: New Zealand
EHR: Electronic Health Record
